# Scar and short-term outcomes after transverse versus modified Henry incisions for volar plating of selected distal radius fractures: a retrospective comparative study

**DOI:** 10.3389/fsurg.2026.1898797

**Published:** 2026-09-14

**Authors:** Xing Zhao, Boxiong Gong, Jian Yang, Feng Shang, Xiaorui Zhou, Xi Wang, Meng Zhao, Shengkang Xu

**Affiliations:** 1 Department of Orthopedics and Trauma, Taihe Hospital, Hubei University of Medicine, Shiyan, Hubei, China; 2 Department of Orthopaedics and Trauma, Beijing Jishuitan Hospital, Capital Medical University, Beijing, China; 3 Department of Nursing, Taihe Hospital, Hubei University of Medicine, Shiyan, Hubei, China

**Keywords:** distal radius fracture, minimally invasive surgery, modified Henry approach, scar assessment, transverse incision, volar locking plate

## Abstract

**Background:**

Volar locking plate fixation is widely used for distal radius fractures (DRFs), but visible scarring and incision-related morbidity remain important patient-centred concerns. This exploratory retrospective study compared scar outcomes and selected short-term clinical outcomes between a single palmar lateral transverse incision and the modified Henry approach in surgically treated DRFs selected for limited volar exposure.

**Methods:**

This retrospective comparative study included 77 of 86 potentially eligible patients with DRFs who underwent volar locking plate fixation between January 2016 and December 2024; nine patients were excluded because of loss to follow-up and/or incomplete outcome data. Thirty-two patients were treated through a single transverse incision, and 45 through the modified Henry approach. Outcomes included tourniquet time, incision length, Lidström radiographic grade, DASH score, POSAS at final follow-up, and complications. Analyses were unadjusted, and Holm correction was applied across seven outcome comparisons.

**Results:**

No transverse-incision procedure required incision extension or conversion to the modified Henry approach, and no major complication occurred. No statistically significant between-group differences were observed in follow-up duration, tourniquet time, dichotomised Lidström grade, or DASH score (all unadjusted *P* > 0.05). The transverse-incision group had shorter incisions (*P* < 0.001; Holm-adjusted *P* < 0.007) and lower PSAS scores (*P* = 0.002; Holm-adjusted *P* = 0.012). The OSAS difference was significant before, but not after, multiplicity correction (unadjusted *P* = 0.032; Holm-adjusted *P* = 0.160).

**Conclusions:**

In this selected cohort, the transverse incision was associated with shorter incisions and lower patient-reported scar scores. The observer-reported scar difference did not remain significant after multiplicity correction. These findings suggest that the transverse incision may offer a scar-related advantage in appropriately selected fractures. The retrospective design and differences in fracture complexity warrant cautious interpretation and prospective confirmation.

## Background

Distal radius fractures (DRFs), defined as fractures within 3 cm of the radiocarpal joint, are among the most common injuries in orthopaedic practice and account for approximately 18% of fractures worldwide (1). Because they involve the wrist, these fractures can cause marked functional limitation and prolonged disability if treatment is suboptimal. Earlier treatment strategies, including cast immobilisation, percutaneous Kirschner-wire fixation, and external fixation, have been associated with secondary displacement, malunion, persistent pain, reduced grip strength, and long-term functional deficits, particularly in unstable fracture patterns (2, 3). These limitations have gradually shifted treatment decisions towards operative fixation for displaced or unstable DRFs (2, 4).

The introduction of volar locking plate systems has advanced the surgical management of DRFs. By providing angular stability, including in osteoporotic bone, these implants facilitate reduction of dorsally displaced fragments and support earlier functional rehabilitation (5). Volar plate fixation through a palmar approach has therefore become a cornerstone of contemporary wrist trauma surgery. Nevertheless, procedure-related soft-tissue complications, most notably tendon irritation, and dissatisfaction with scar appearance remain clinically relevant concerns (6–8). Cosmetic dissatisfaction may persist even when radiographic alignment and functional recovery are acceptable. This underscores the increasing importance of patient-centred outcomes in fracture care.

The modified Henry approach is widely regarded as the standard palmar approach for volar plate fixation of DRFs (9). It uses a longitudinal incision along the flexor carpi radialis tendon, providing reliable exposure of the pronator quadratus and fracture site (10). Although this exposure facilitates visualisation and reduction, the relatively long incision and broader soft-tissue dissection may increase incision-related morbidity and reduce cosmetic satisfaction. Prior minimally invasive modifications include dual-incision plate insertion, longitudinal mini-incisions, and three-point positioning techniques (11–14). These approaches differ in incision configuration and technical demands.

In routine surgical practice, we aimed to refine the balance between adequate exposure and incision minimalism. Guided by local anatomy and the orientation of natural wrist creases, we adopted a single palmar lateral transverse incision for volar locking plate fixation. The approach was intended to preserve the biomechanical advantages of volar plating while improving scar appearance and patient satisfaction.

Accordingly, this exploratory retrospective study compared scar-related and selected short-term outcomes between the single palmar lateral transverse incision and the modified Henry approach. We hypothesised that the transverse incision would be associated with more favourable scar outcomes in fractures judged suitable for limited volar exposure.

## Methods

### Study design and ethical approval

This retrospective comparative study was conducted at Taihe Hospital, Shiyan, Hubei Province, China. The Ethics Committee of Shiyan Taihe Hospital approved the study (No. 2023KS51), and all procedures complied with the Declaration of Helsinki. Because the study was retrospective and used anonymised routine clinical data, the Ethics Committee waived the requirement for informed consent for participation. Written informed consent for surgical treatment was obtained from all patients before surgery.

### Patient selection

Patients with distal radius fractures treated surgically between January 2016 and December 2024 were screened. Inclusion criteria were: (1) diagnosis of DRF confirmed by plain radiography or computed tomography; (2) AO type A2–A3, B1–B3, or C1 fracture that met the morphology restrictions described below; (3) treatment with volar locking plate fixation through either a single palmar lateral transverse incision or the modified Henry approach; (4) age ≥18 years; and (5) informed consent for operative management. The decision to operate was made by the treating team for a displaced or unstable fracture after clinical and imaging review.

Exclusion criteria were open fractures or associated vascular or nerve injuries, concomitant fractures of the ipsilateral upper limb, surgery delayed by more than 2 weeks after injury, pathological fractures, and pre-existing dysfunction of the affected upper limb. Morphological exclusions for the limited approach included marked metaphyseal comminution, multifragmentary intra-articular involvement, a dorsal lunate facet fragment requiring direct exposure, a four-part fracture, substantial metaphyseal bone loss, or any pattern judged to require broad articular visualisation.

### Patient grouping and baseline characteristics

Eighty-six potentially eligible patients were identified. Nine were excluded from the final analysis because of loss to follow-up and/or incomplete outcome data, leaving 77 patients: 32 treated using the single palmar lateral transverse incision (11 males and 21 females; mean age, 54.52 ± 12.91 years) and 45 through the modified Henry approach (16 males and 29 females; mean age, 52.47 ± 15.52 years). Although no baseline comparison reached statistical significance, type C fractures were less frequent in the transverse group than in the modified Henry group [5/32 [15.6%] versus 15/45 [33.3%]] (Table 1).

**Table 1:** Baseline demographic and clinical characteristics.

Variable	Single transverse incision (*n* = 32)	Modified Henry approach (*n* = 45)	Test statistic	*P* value	Effect size
Sex (male/female)	11/21	16/29	*χ*^2^ = 0.01	0.92	Cramér's V = 0.01
Age (years)	54.52 ± 12.91	52.47 ± 15.52	t = 0.63	0.53	Cohen's d = 0.14
Mechanism (low/high energy)	21/11	33/12	χ^2^ = 0.53	0.46	Cramér's V = 0.08
Side (dominant/non-dominant)	27/5	32/13	χ^2^ = 2.58	0.11	Cramér's V = 0.18
Injury-to-surgery interval (days)	2.82 ± 1.19	2.61 ± 1.35	t = 0.69	0.49	Cohen's d = 0.16
AO type (A/B/C)	7/20/5	12/18/15	χ^2^ = 4.21	0.12	Cramér's V = 0.23

Data are presented as mean ± standard deviation or number of patients.

### Preoperative and operative procedures

All patients underwent preoperative wrist radiographs and computed tomography with three-dimensional reconstruction when needed to characterise fracture morphology and plan fixation. Initial closed reduction and temporary splint immobilisation were applied, and surgery was performed after swelling allowed safe wound closure. Prophylactic antibiotics were administered 30 min before incision. A single surgical team performed all procedures. The choice of approach was made by the treating surgeon and was not randomised.

### Selection of surgical approach

The transverse incision was considered for AO A2–A3 and selected B1–B3 or C1 fractures after systematic preoperative imaging review using the features listed in Table 2. For B3 and C1 fractures, suitability was determined from the complete fragment configuration, metaphyseal comminution, bone loss, and the exposure required for reduction and fixation. The transverse approach was selected when stable reduction, plate positioning, and screw placement were expected to be achievable through limited volar exposure.

**Table 2:** Preoperative imaging checklist for the transverse approach.

Imaging domain	Transverse incision considered only when
AO pattern	A2–A3, or B1–B3/C1 patterns with favourable morphology after comprehensive imaging review.
Articular morphology	No multifragmentary pattern, dorsal lunate facet fragment requiring direct exposure, four-part configuration, or need to visualise multiple fragments.
Metaphysis	No marked metaphyseal comminution or substantial metaphyseal bone loss.
Reduction and fixation	Reduction, plate positioning, and screw placement were expected to be achievable under fluoroscopy through limited exposure.
Integrated decision	Use the transverse approach when reduction and fixation are expected to be achievable through limited exposure; retain readiness to extend or convert when wider visualisation is required.

The exposure-escalation protocol prioritised reduction and implant safety. If reduction or plate insertion was difficult, the radial or distal pronator quadratus was partially released, with full release if required. If acceptable reduction, plate position, or screw trajectory could not be confirmed, the incision was to be extended or converted to the modified Henry approach.

No minimum procedural volume was prespecified for use of the transverse approach. The technique should be used only by surgeons proficient in standard volar plating who can extend or convert the exposure when necessary.

For the single transverse incision approach, a 2–3 cm incision was made along the wrist flexion crease on the palmar radial side. After the palmaris longus and flexor carpi radialis (FCR) tendons were identified, the FCR sheath was opened and blunt dissection proceeded while the median nerve was directly identified and protected. The working interval was continued to the pronator quadratus, which was exposed with gentle soft-tissue handling. Reduction was performed under fluoroscopy, and a volar locking plate was inserted beneath the pronator quadratus and fixed with sequential locking screws.

In the modified Henry approach, a longitudinal incision was made along the radial aspect of the forearm. Dissection proceeded between the flexor carpi radialis tendon and the radial artery to expose the pronator quadratus. The pronator quadratus was incised to permit direct visualisation and fracture reduction, and plate positioning and fixation were performed according to standard surgical protocols.

### Postoperative care and follow-up

After surgery, all patients received antibiotic prophylaxis for 1–2 days. Early active finger motion was encouraged immediately, and progressive wrist mobilisation began approximately 48 h postoperatively. Rehabilitation intensity was increased according to radiographic evidence of fracture stability, with weight-bearing and strengthening exercises typically introduced after 6 weeks.

Perioperative variables included tourniquet time, incision length, and postoperative complications. Postoperative alignment was graded on postoperative radiographs using the Lidström system (15) as excellent, good, fair, or poor; for analysis, excellent/good grades were combined as satisfactory and fair/poor grades as unsatisfactory. Scar quality was assessed at each patient's final follow-up using POSAS (16). OSAS was assessed independently by two attending-level orthopaedic physicians; disagreements were adjudicated by a senior chief orthopaedic physician. Because incision orientation was visible, assessors could not be blinded. Upper-limb function was assessed using DASH (17).

### Statistical analysis

Statistical analyses were performed using IBM SPSS Statistics version 24.0 (IBM Corp., Armonk, NY, USA). Data distribution was assessed with the Shapiro–Wilk test. Continuous variables are presented as mean ± standard deviation and were compared using independent-samples t-tests. Categorical variables were analysed using the chi-square test or Fisher's exact test, as appropriate. Mean differences and 95% confidence intervals are reported with effect sizes. To address multiplicity across the seven outcomes in Table 3, Holm-adjusted *P* values are reported alongside unadjusted two-sided *P* values. No *a priori* sample size calculation was performed. *Post hoc* power was not calculated; confidence intervals were used to show estimate precision. No propensity-score or multivariable adjustment was performed, and the between-group estimates therefore remain unadjusted.

**Table 3:** Intraoperative and postoperative outcomes.

Variable	Transverse (*n* = 32)	Modified Henry (*n* = 45)	Statistic	*P* (raw; Holm)	Difference (95% CI); effect size
Tourniquet time (min)	57.38 ± 10.57	53.93 ± 8.41	t = 1.59	0.12 (0.480)	3.45 (−0.87 to 7.77); d = 0.36
Incision length (cm)	2.48 ± 0.97	5.37 ± 1.53	t = −9.47	<0.001 (<0.007)	−2.89 (−3.50 to −2.28); d = 2.20
Follow-up duration (months)	13.28 ± 3.51 (range, 11.9–28.5)	12.94 ± 3.72 (range, 11.6–29.8)	t = 0.41	0.68 (1.000)	0.34 (−1.31 to 1.99); d = 0.09
Lidström grade (excellent/good vs fair/poor)	31/1	43/2	χ^2^ = 0.08	0.76 (1.000)	1.3% (−7.2% to 9.8%); V = 0.03
OSAS score (6–60, lower is better)	8.11 ± 4.27	10.59 ± 5.52	t = −2.18	0.032 (0.160)	−2.48 (−4.75 to −0.21); d = 0.50
PSAS score (6–60, lower is better)	10.13 ± 5.57	14.68 ± 6.39	t = −3.27	0.002 (0.012)	−4.55 (−7.32 to −1.78); d = 0.75
DASH score (0–100, lower is better)	7.47 ± 11.65	8.63 ± 10.29	t = −0.47	0.64 (1.000)	−1.16 (−6.08 to 3.76); d = 0.11

Data are presented as mean ± standard deviation or number of patients. Mean and risk differences are defined as transverse minus modified Henry. The *P*-value column reports unadjusted and Holm-adjusted values for the seven outcome comparisons. Lidström grades are shown as satisfactory (excellent/good) versus unsatisfactory (fair/poor). OSAS and PSAS each ranged from 6 to 60, with lower scores indicating better scar quality. DASH scores ranged from 0 to 100, with lower scores indicating better function.

## Results

Of 86 potentially eligible patients, nine were excluded because of loss to follow-up and/or incomplete outcome data. Seventy-seven patients were included in the final analysis: 32 in the single transverse incision group and 45 in the modified Henry group. Type C fractures accounted for 15.6% and 33.3% of the groups, respectively (*P* = 0.12 for the broad AO distribution) (Table 1).

At final follow-up, mean follow-up duration was 13.28 ± 3.51 months in the transverse group and 12.94 ± 3.72 months in the modified Henry group; the corresponding ranges were 11.9–28.5 months and 11.6–29.8 months, respectively (mean difference, 0.34 months; 95% CI, −1.31 to 1.99; unadjusted *P* = 0.68). Mean tourniquet time was 57.38 ± 10.57 min and 53.93 ± 8.41 min, respectively (mean difference, 3.45 min; 95% CI, −0.87 to 7.77; unadjusted *P* = 0.12).

A satisfactory Lidström grade (excellent/good) was recorded in 31 of 32 patients in the transverse group and 43 of 45 in the modified Henry group (risk difference, 1.3 percentage points; 95% CI, −7.2 to 9.8; unadjusted *P* = 0.76). DASH scores were 7.47 ± 11.65 and 8.63 ± 10.29, respectively (mean difference, −1.16; 95% CI, −6.08 to 3.76; unadjusted *P* = 0.64). The results are summarised in Table 3.

Incision length was shorter in the transverse group than in the modified Henry group (2.48 ± 0.97 cm versus 5.37 ± 1.53 cm; mean difference, −2.89 cm; 95% CI, −3.50 to −2.28; unadjusted *P* < 0.001; Holm-adjusted *P* < 0.007). PSAS scores were also lower (10.13 ± 5.57 versus 14.68 ± 6.39; mean difference, −4.55; 95% CI, −7.32 to −1.78; unadjusted *P* = 0.002; Holm-adjusted *P* = 0.012). OSAS scores were lower before multiplicity correction (8.11 ± 4.27 versus 10.59 ± 5.52; mean difference, −2.48; 95% CI, −4.75 to −0.21; unadjusted *P* = 0.032), but this comparison was not significant after Holm correction (adjusted *P* = 0.160) (Table 3).

Figures 1, 2 illustrate the operative technique and postoperative follow-up of two patients treated through the transverse incision.

**Figure 1:**
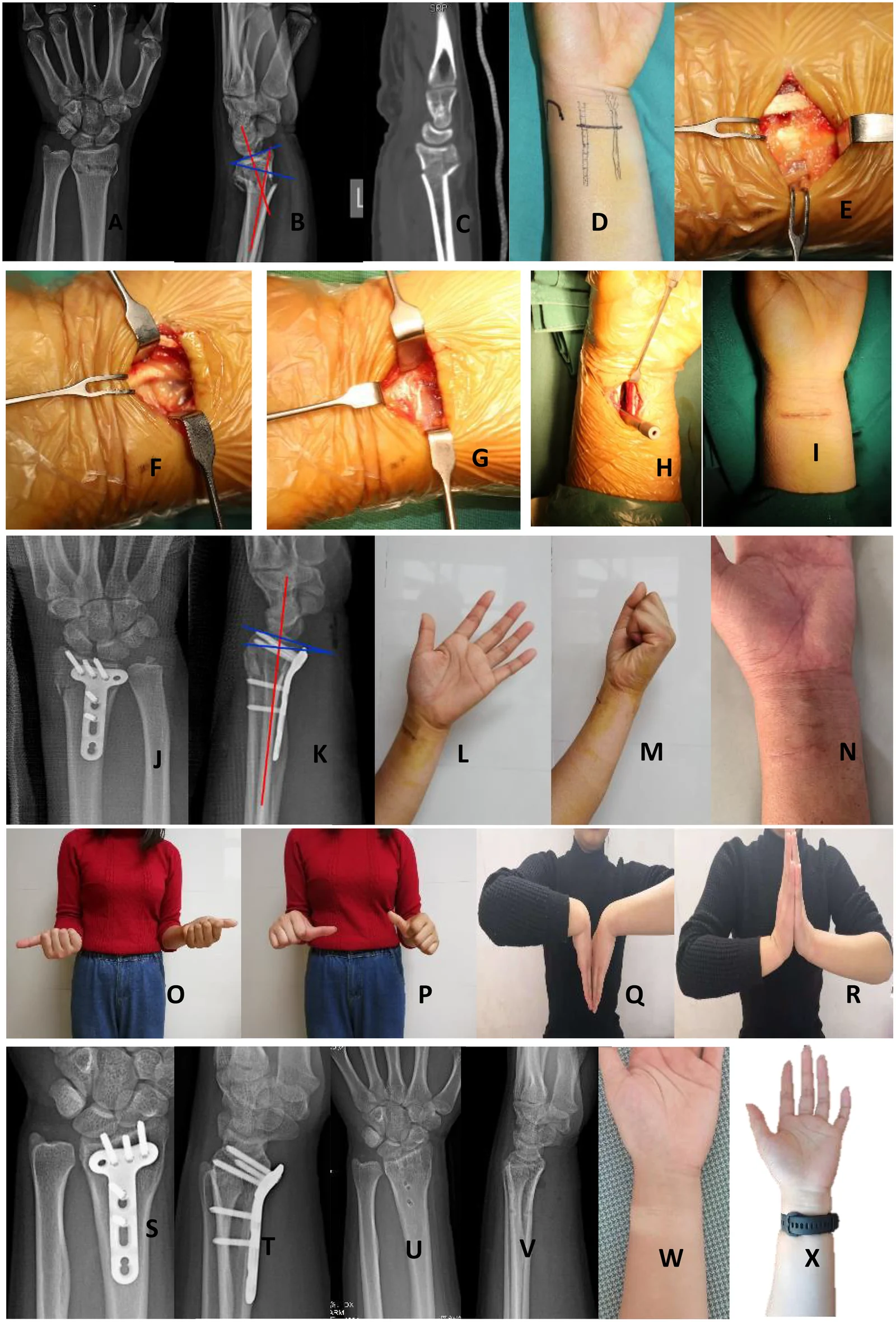
Illustrative case treated with a single transverse palmar incision. A 19-year-old woman sustained a left distal radius fracture after a fall. Preoperative radiographs showed radial shortening **(A)** and dorsal displacement **(B)**, and computed tomography showed intra-articular involvement **(C)** Surface landmarks and the planned incision were marked **(D)** Through a single transverse palmar incision, the palmaris longus and FCR tendons were identified, the FCR sheath was opened, and the median nerve was directly identified and protected during blunt dissection **(E,F)**. The pronator quadratus was then exposed **(G)**, followed by reduction, volar locking plate fixation **(H)**, and closure **(I)**. Subsequent panels illustrate postoperative alignment, early motion, union, planned elective implant removal, and scar maturation **(J–X)**.

**Figure 2:**
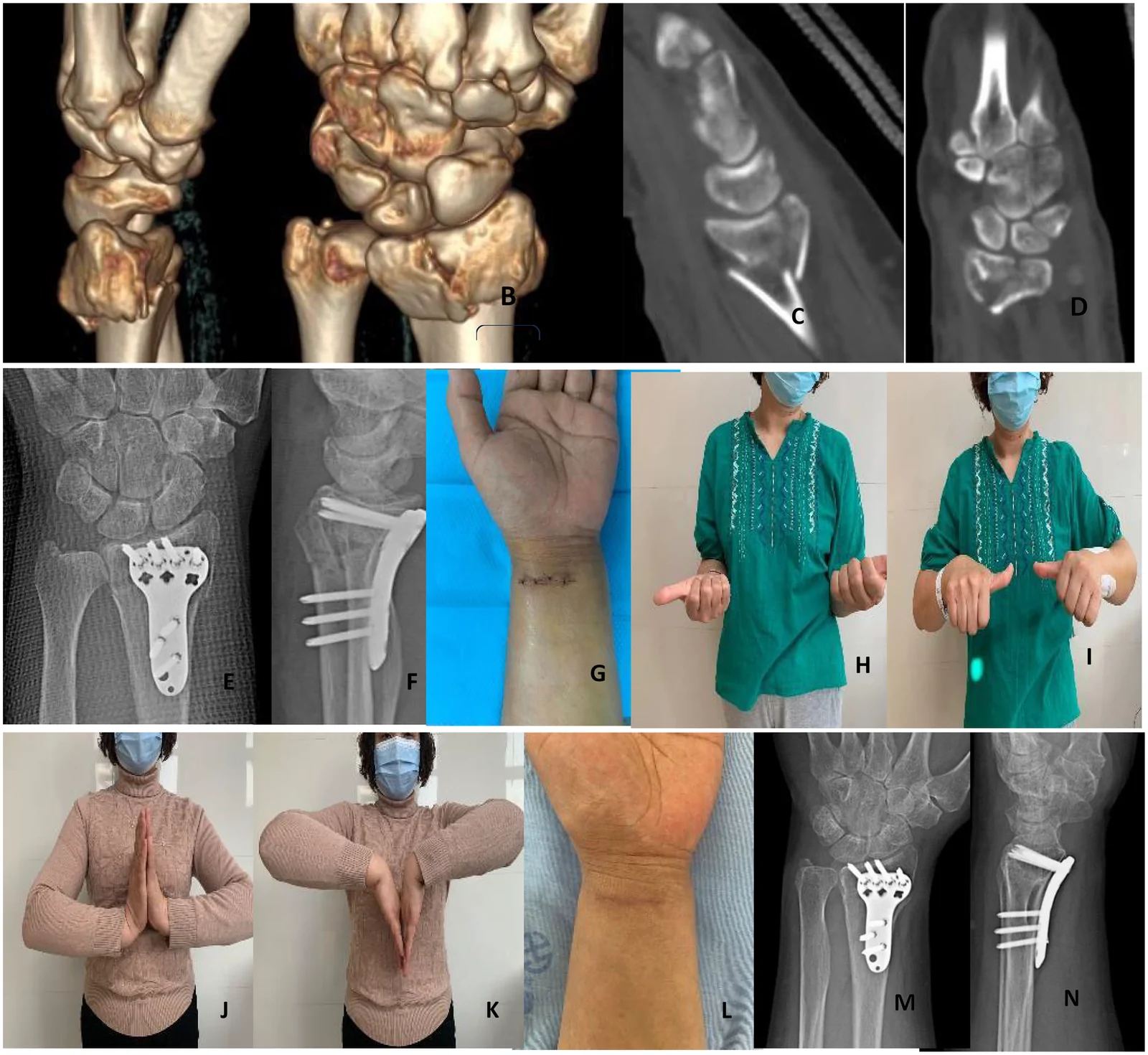
Illustrative case treated with a single transverse palmar incision using a mini volar plate. A 58-year-old woman sustained a left distal radius fracture after a fall. Preoperative computed tomography showed dorsal displacement with intra-articular involvement **(A-D)**. The fracture was treated through a single transverse palmar incision using a low-profile volar locking plate. Postoperative radiographs showed restoration of radial height and volar tilt **(E,F)**. Subsequent panels illustrate the early postoperative scar **(G)**, early forearm rotation **(H,I)**, later wrist motion **(J,K)**, scar appearance **(L)**, and fracture union **(M,N)**.

All transverse-incision procedures were completed without incision extension or conversion to the modified Henry approach. One patient in the transverse group developed transient numbness in the palmar thenar region, which resolved within 1 month with conservative management. No deep infection, nonunion, implant failure, screw loosening, unplanned reoperation, or other major complication occurred in either group. The implant removal shown in Figure 1 was a planned elective procedure after fracture union.

## Discussion

The main finding was that, among patients selected for volar plate fixation, the transverse approach was associated with a shorter incision and lower patient-reported scar scores than the modified Henry approach. The observer-reported scar difference was significant in the unadjusted analysis but not after multiplicity correction. No statistically significant between-group differences were observed in the assessed Lidström grade or DASH score. These results support the transverse incision as a cosmetically favourable option for appropriately selected fractures, while the retrospective design and imbalance in fracture complexity warrant cautious interpretation.

### Cosmetic outcomes and scar perception

Postoperative scar appearance is a relevant patient-centred outcome in wrist trauma surgery (16, 18). The transverse group had shorter incisions and lower PSAS scores after Holm correction. The OSAS difference was supported by the unadjusted analysis but not after correction, and observer bias cannot be excluded because incision orientation was visible. The patient-reported finding is therefore the more robust scar-related signal in this study.

Incisions placed parallel to natural skin tension lines, especially along wrist flexion creases, may reduce mechanical stress during healing and promote more favourable scar maturation (19–21). From a mechanobiological perspective, lower tension across a healing incision may attenuate fibroblast activation and disorganised collagen deposition, both of which are implicated in hypertrophic scarring (18).

Serial photographs in Figures 1, 2 illustrate progressive scar maturation along the wrist crease during follow-up.

### Position relative to other minimally invasive approaches

The present technique builds on minimally invasive volar plating strategies for distal radius fractures. Unlike previously described dual-incision systems, longitudinal mini-incisions, and three-point positioning approaches (11–14), it uses a single transverse wrist-crease incision. This configuration combines limited volar exposure with alignment along the natural wrist crease and permits scar outcomes to be evaluated using the patient- and observer-reported components of POSAS. These features define its practical distinction among minimally invasive volar approaches.

### Functional and clinical outcomes

Volar locking plate fixation provides stable fixation and supports early mobilisation (5, 24). In this study, both groups had low mean DASH scores at final follow-up, with no statistically significant between-group difference. Together with the early wrist and finger motion observed during follow-up, these findings indicate favourable short-term upper-limb recovery after both approaches. Future prospective studies incorporating PRWE, objective wrist range of motion, grip strength, and pain scores would provide a more comprehensive functional assessment.

Early wrist and finger motion in the illustrative cases further demonstrates the feasibility of the postoperative mobilisation protocol after the transverse approach.

### Indications and technical considerations

The transverse approach was primarily used for AO A2–A3 and selected B1–B3 or C1 fractures without marked metaphyseal comminution, a dorsal lunate facet fragment requiring direct exposure, four-part configuration, substantial bone loss, or a need to visualise multiple articular fragments. These features provide a practical imaging-based framework for identifying fractures amenable to limited volar exposure. For B3 and C1 fractures, suitability should be determined from the complete fragment configuration and the exposure required for reduction rather than from AO subtype alone.

Satisfactory Lidström grades were achieved in 96.9% of patients treated through the transverse incision and 95.6% treated through the modified Henry approach, with no statistically significant between-group difference. These findings indicate that the transverse approach achieved a high rate of satisfactory postoperative alignment within the selected indications. The higher proportion of type C fractures in the modified Henry group is consistent with use of the broader exposure for more complex patterns. Together, the findings support a selective strategy in which the transverse approach offers a scar-related advantage for fractures amenable to limited exposure, whereas the modified Henry approach remains appropriate when wider visualisation is required.

One patient experienced transient irritation of the palmar cutaneous branch, but such complications are not unique to transverse incisions. Previous studies suggest that nerve irritation is more closely related to traction, soft-tissue handling, and anatomical variation than to incision orientation alone (22, 23). This observation reinforces the importance of meticulous dissection and anatomical awareness regardless of incision design.

From a clinical perspective, the transverse incision may also support preoperative counselling. Aligning the surgical approach with patient expectations regarding scar appearance may improve overall treatment satisfaction without changing established fixation principles.

Several limitations should be considered when interpreting these findings. First, the retrospective single-centre design and surgeon-guided approach selection may have introduced selection bias, particularly because type C fractures were more frequent in the modified Henry group; the between-group estimates were unadjusted, with no propensity-score, multivariable, or A/B-restricted sensitivity analysis. Patient-flow data combined loss to follow-up and incomplete outcome data rather than reporting these reasons separately. Second, the imaging checklist reflects the selection strategy used in clinical practice and requires prospective validation with prespecified quantitative thresholds. Third, radiographic evaluation relied on the dichotomised Lidström grade rather than the complete four-category distribution and did not include a complete set of wrist-specific measurements. Fourth, POSAS and DASH were assessed at the final follow-up visit, which occurred at variable times, and the follow-up ranges were 11.9–28.5 months in the transverse group and 11.6–29.8 months in the modified Henry group. OSAS could not be blinded because incision orientation was visible; although two attending-level physicians assessed scars independently with senior adjudication, inter-rater reliability was not quantified. Fifth, functional assessment used DASH without PRWE, objective wrist motion, grip strength, or pain scores, and the study did not include a nonoperative comparison group. Sixth, the modest sample size and absence of an *a priori* sample-size or noninferiority framework limited the precision of nonsignificant comparisons. Seventh, surgeon-specific case volume and learning-curve outcomes were not evaluated. Within these boundaries, the study provides clinically relevant evidence of scar-related advantages and favourable short-term alignment and functional outcomes in appropriately selected patients. Future multicentre prospective studies with standardised follow-up, comprehensive wrist-specific outcomes, and adjusted analyses should confirm and extend these findings.

## Conclusions

In appropriately selected distal radius fractures, the single palmar lateral transverse incision was associated with shorter incisions and lower patient-reported scar scores than the modified Henry approach; the observer-reported scar difference did not remain significant after multiplicity correction. The transverse approach may represent a practical option with a patient-reported scar advantage in appropriately selected fractures. Multicentre prospective studies using standardised wrist-specific outcomes and adjusted analyses should further validate these findings.

## Data Availability

The clinical datasets used and/or analysed during the current study are not publicly available because of privacy protection and institutional restrictions. They are available from the corresponding author on reasonable request.
